# Cardiac Amyloidosis: A Comprehensive Review of Pathophysiology, Diagnostic Approach, Applications of Artificial Intelligence, and Management Strategies

**DOI:** 10.7759/cureus.63673

**Published:** 2024-07-02

**Authors:** Md Ripon Ahammed, Fariha Noor Ananya

**Affiliations:** 1 Internal Medicine, Icahn School of Medicine at Mount Sinai/New York City Health and Hospitals Queens, New York City, USA; 2 Internal Medicine, Dhaka Medical College and Hospital, Dhaka, BGD

**Keywords:** artifical intelligence, al cardiac amyloidosis, transthyretin amyloid cardiomyopathy, ttr cardiac amyloidosis, cardiac amylodosis

## Abstract

Cardiac amyloidosis (CA) is a serious and often fatal condition caused by the accumulation of amyloid fibrils in the heart, leading to progressive heart failure. It involves the misfolding of normally soluble proteins into insoluble amyloid fibrils, with transthyretin and light-chain amyloidosis being the most common forms affecting the heart. Advances in diagnostics, especially cardiac magnetic resonance imaging and non-invasive techniques, have improved early detection and disease management. Artificial intelligence has emerged as a diagnostic tool for cardiac amyloidosis, improving accuracy and enabling earlier intervention through advanced imaging analysis and pattern recognition. Management strategies include volume control, specific pharmacotherapies like tafamidis, and addressing arrhythmias and advanced heart failure. However, further research is needed for novel therapeutic approaches, the long-term effectiveness of emerging treatments, and the optimization of artificial intelligence applications in clinical practice for better patient outcomes. The article aims to provide an overview of CA, outlining its pathophysiology, diagnostic advancements, the role of artificial intelligence, management strategies, and the need for further research.

## Introduction and background

Amyloidosis is a disease of multisystems in which extracellular accumulation of amyloid (a material detected on Congo red staining followed by apple-green birefringence under polarized light) damages multiple organs. It is caused by normal soluble proteins becoming insoluble amyloid fibrils by conformational change [1]. Nine of the approximately thirty amyloid precursor proteins significantly impact the heart. These include transthyretin (TTR), which causes transthyretin amyloidosis (ATTR) in both familial/hereditary ATTR (variant ATTR [ATTRv]) and wild-type/non-hereditary (ATTRwt) forms, and light chains, which causes AL amyloidosis. Other less common forms include fibrinogen A-α, gelsolin, β2-microglobulin, apolipoproteins, and serum component A. The two leading causes of amyloid cardiomyopathy (CM) are AL-CM and ATTR-CM, with ATTR-CM recently becoming more common in developed nations [2]. ATTRv amyloidosis is caused by destabilizing mutations in the TTR gene, which can arise in younger adults, whereas the more common ATTRwt amyloidosis is linked to aging [3]. By February 2023, the Cardiac Amyloidosis Registry Study (CARS) had enrolled 1415 patients from 20 US centers. 82% of the patients had ATTR cardiac amyloidosis, with 71% of them having the wild-type and 68% with the p.V142I mutation. In addition, females were more prevalent in the AL group (39%) than in the ATTR group (13%) [4]. Two prospective studies identified 13% and 18% prevalence for ATTRwt among individuals over 60 with heart failure with preserved ejection fraction (HFpEF) and wall thickness greater than 12 mm. Furthermore, ATTR-CM prevalence was noted at 9% in patients having heart failure with reduced ejection fraction (HFrEF) of unknown etiology. The studies excluded participants with other potential heart failure origins, like significant valvulopathies or previous coronary diseases, suggesting a possible underrepresentation of ATTR-CM prevalence [5-7]. Cardiac magnetic resonance (CMR) imaging and an image-based methodology that permits diagnosis without tissue biopsy in roughly 70% of cases have led to a notable increase in ATTR-CM diagnosis. As a result, more patients are recognized earlier in the disease, which is associated with improved heart structure and preservation of function [8]. A 20-year UK study found that diagnoses of ATTR-CM, especially wild-type, were much higher, with almost 60% of recent diagnoses (2017-2021) being at an early stage [9]. This pattern has improved our knowledge of how diseases progress and the variables affecting their clinical variability. This review aims to discuss the pathophysiology of CA, management strategies, and diagnostic approaches, including the current advancement of artificial intelligence in the field.

## Review

Pathophysiology

TTR, a plasma protein primarily generated in the liver, choroid plexuses, and retina, is deposited to cause ATTR. TTR is responsible for transporting retinol-bound protein and thyroxine. Age-related factors in ATTRwt or genetic mutations in ATTRv cause TTR to split into dimers and monomers. For still unclear reasons, these monomers combine to create fibrils and deposit primarily in the heart and nervous tissue [2]. In vitro, when the tetrameric structure of TTR becomes unstable, the protein breaks down into monomers and dimers, resulting in misfolding into an inactive conformation. This process is crucial for the formation of amyloid fibrils. Simulations of molecular dynamics show that dissociation of the tetramer is a multi-step process, with the most notable energy barrier occurring during the transition. Improper posttranslational modifications, altered proteostasis, and metal cations play a role in the instability of the TTRwt structure. TTR monomers may misfold, forming a stable "misfolded state" prone to aggregation. Misfolded TTR monomers interact to form dimers, which then combine to create spherical hexamers, serving as building blocks for cytotoxic oligomers. The amyloid formation kinetics consist of three phases: nucleation, growth, and saturation [10]. Prior research on ATTRwt has focused chiefly on its effects on cardiac tissue. Research on cardiac fibroblasts has shown that the presence of TTR in the extracellular matrix of the tissue may impact the genetic composition, capabilities, and expression of these cells. Transcriptional sequencing and cytokine proteomic analysis revealed increased inflammatory genes that may exacerbate cardiac inflammation and fibrosis. Additionally, TTRwt amyloid toxicity causes mitochondrial dysfunction, oxidative stress in cardiomyocytes, and disruptions in calcium levels and cycling, which may result in cardiac dysfunction [11].

Over 150 mutations have been found, most linked to ATTRv. The Val122Ile is the most prevalent form in the USA, affecting African and Caribbean descent. The disease onset is late, usually after 60, and patients accumulate myocardial amyloid. Men are more likely to be affected than women. The second most common type of ATTRv genotype is Thr60Ala amyloidosis, predominantly affecting Caucasians. Epigenetics may determine which carriers will develop ATTRv disease. Genetic counseling should accompany ATTRv testing decisions. To determine the effectiveness and financial feasibility of genetic testing, early intervention, and surveillance, further research is necessary [12]. A study in Bologna, Italy, analyzed 325 patients with ATTRv mutations between 1984 and 2022. TTR gene variants were identified in the study, with the most prevalent ones being Ile68Leu (41.8%), Val30Met (19%), and Glu89Gln (10%). After 51 months, 38.3% of patients died, and 11.5% developed ATTRv. Key factors influencing survival included age at diagnosis, NYHA class III, left ventricular ejection fraction (LVEF), and disease-modifying therapy. Family screening programs are essential for early detection and managing ATTRv to reduce mortality [13]. The Transthyretin Amyloidosis Outcomes Survey (THAOS) registry, initiated in 2007, is the largest and oldest global study on ATTR amyloidosis. The most recent update includes 3,779 symptomatic patients and 1,830 asymptomatic TTR mutation carriers from 23 different countries. Among the symptomatic individuals, 16.6% had a mixed phenotype, 40.7% had a cardiac phenotype, and 40.1% had a neurologic phenotype [14].

Clinical manifestation and diagnosis

A majority of patients initially present with nonspecific symptoms such as fatigue, weight loss, and edema, followed by the development of more focused symptoms [15]. The delayed diagnosis of cardiac amyloidosis is often caused by its numerous nonspecific symptoms [16]. For instance, upon diagnosis of AL amyloidosis, around 67% of patients have kidney involvement, and 50% have heart involvement, with up to 90% of patients experiencing heart involvement as the disease progresses [17,18]. Arrhythmia and heart failure account for about 75% of deaths from AL amyloidosis [16].

Red flags and clinical clues associated with cardiac amyloidosis can be broadly classified into two categories: extracardiac manifestations (such as history of proteinuria, autonomic/peripheral neuropathy, spinal stenosis, carpal tunnel syndrome, knee or hip replacement, or previous shoulder surgery) and cardiac features (such as left ventricular hypertrophy without the presence of cardiac valvular disease or hypertension, diastolic abnormality, symptoms of heart failure (HF), atrial fibrillation [AF], conduction defect, raised cardiac biomarkers) [19]. Significant overlap exists in the clinical, radiological, and electrocardiographic characteristics of AL and ATTR amyloidosis, making cardiac symptoms insufficient to differentiate the two conditions. Pathognomonic extracardiac signs of AL amyloidosis include periorbital purpura secondary to increased fragility of capillary, acquired Factor X insufficiency, and enlarged tongue/submandibular gland due to involvement of soft tissue. Conversely, ATTR amyloidosis is exclusive to musculoskeletal symptoms such as spontaneous rupture of the biceps tendon and spinal stenosis. Peripheral neuropathy, orthostatic hypotension, GI involvement, and CM are frequent organ systems impacted by both ATTR and AL amyloidosis [19,20]. 

ATTR cardiac amyloidosis (CA) follows a more gradual and slow-developing course compared to AL-CA. Patients with cardiac involvement in ATTR have a considerably better prognosis, with an average of five more years of survival compared to those with cardiac involvement in AL amyloidosis. However, the initial slow progression of ATTR-CA often leads to under-recognition. [21]. When diagnosing cardiac amyloidosis (CA), the following criteria are considered: unexplained left ventricular (LV) thickness (≥12 mm), specific echocardiography findings, and a multiparametric echocardiographic score. To confirm CA using echocardiography, at least two of the following must be present: diastolic dysfunction grade 2 or more, decreased tissue Doppler s', e', and a' wave velocities (<5 cm/s), and reduction in LV global longitudinal strain (LGLS) less than < −15%. Additionally, a multiparametric echocardiographic score of ≥8 is used, which includes criteria such as relative LV wall thickness >0.6 (3 points), LGLS absolute value of ≤ −13% (1 point), Doppler E/e' wave velocities >11 (1 point), TAPSE (tricuspid annular plane systolic excursion) of ≤19 mm (2 points), and systolic longitudinal strain apex to base ratio >2.9 (3 points). For cardiac magnetic resonance (CMR), both diffuse transmural or subendocardial late gadolinium enhancement (LGE) and abnormal gadolinium kinetics must be present. An extracellular volume (ECV) ≥0.40% is strongly indicative but unessential for diagnosis [22].

Bone scintigraphy, using 99mTechnetium (Tc) labeled tracers such as 3,3-diphosphono-1,2-propanodicarboxylic acid (DPD), hydroxymethylene diphosphonate (HMDP), and pyrophosphate (PYP), is an important non-invasive method for diagnosing CA. It can detect intense cardiac uptake, indicating the presence of the disease. This method has high sensitivity and specificity, particularly for ATTR CA, with clinical studies showing sensitivity and specificity of over 90%. This reduces the need for invasive biopsies and allows for early and accurate treatment interventions. Additionally, AI systems further enhance diagnostic accuracy [23]. Bone scintigraphy with technetium 99 m should be performed on patients with suspected CA, either concomitantly with monoclonal protein evaluation or after a negative protein assessment. The results are interpreted in a semi-quantitative way, with Grade 0 showing no tracer myocardial uptake and normal bone uptake, Grade 1 showing lower myocardial uptake compared to bone level, Grade 2 showing equal myocardial and bone uptake, and Grade 3 showing greater myocardial uptake compared to bone. A quantitative analysis of scintigraphy results determining the ratio of tracer uptake between the heart (H) and the contralateral half of the lung (CL) can be applied, with values > 1.5 considered positive. Bone scintigraphy should always be combined with single-photon emission computed tomography (SPECT) imaging to avoid false positive results due to blood pooling in the heart [20].

**Figure 1:**
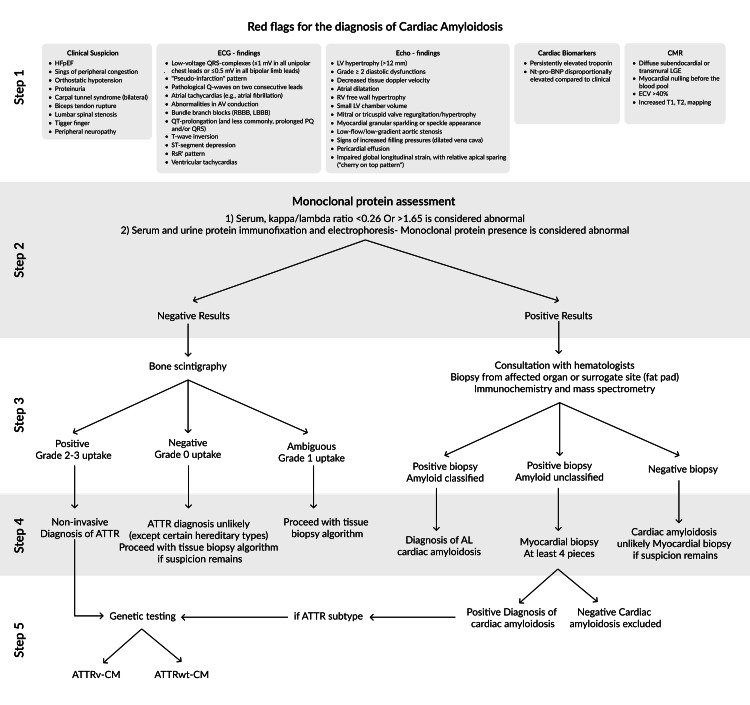
Diagnostic algorithm of cardiac amyloidosis HFpEF: heart failure with preserved ejection fraction; RBBB: right bundle branch block; LBBB: left bundle branch block; LV: left ventricle; NT-proBNP: N-terminal pro-brain natriuretic peptide; LGE: late gadolinium enhancement; ECV: extracellular volume; ATTR: transthyretin amyloidosis; ATTRv-CM: hereditary transthyretin amyloid cardiomyopathy; ATTRw-CM: wild-type transthyretin amyloid cardiomyopathy Figure 1 has been adapted from Briasoulis A, Bampatsias D, Papamichail A, Kuno T, Skoularigis J, Xanthopoulos A, Triposkiadis F. Invasive and Non-Invasive Diagnostic Pathways in the Diagnosis of Cardiac Amyloidosis. Journal of Cardiovascular Development and Disease. 2023; 10(6):256 [20]. Available under the Creative Commons Attribution License (CC BY). [59]

A comprehensive diagnostic algorithm is illustrated in Figure 1 [19,20].

Apolipoprotein AI amyloidosis (AApoAI) and Apolipoprotein AIV amyloidosis (AApoAIV) are rare but increasingly recognized causes of CA, where AApoAI-CA commonly presents with heart failure or dysphonia, with the Arg173Pro variant causing cardiac and laryngeal involvement. AApoAIV-CA is associated with right-sided involvement, thicker right ventricular free wall, greater incidence of tricuspid stenosis, and tricuspid regurgitation. AApoAIV-CA most commonly presents with heart failure and a lower median estimated glomerular filtration rate than AL-CA and TTR-CA. Both AApoAI and AApoAIV have a good prognosis and lower mortality risk than matched patients with AL-amyloidosis [24].

Role of artificial intelligence (AI) on the diagnosis of cardiac amyloidosis

CA diagnosis is challenging due to its high variability and requires expertise. Artificial intelligence (AI), like deep-learning convolutional neural networks, is quickly changing cardiovascular medicine by identifying higher-risk patients and expediting the diagnosis process [25]. Recent studies have explored the role of AI in diagnosing cardiac amyloidosis (CA) through various medical modalities. Tison et al. developed machine learning (ML) algorithms for cardiac parameter evaluation using 36,186 ECGs, achieving an Area Under the Curve (AUC) of 0.86 for predicting CA. However, the study was limited to a single center and normal sinus rhythm [26]. Goto et al. created an AI-ECG model for detecting CA with an AUC of 0.85 to 0.91 using 10,933 ECGs capable of identifying CA before clinical diagnosis. Still, they faced potential false labels due to undiagnosed cases in the control group [27]. Grogan et al. developed an AI tool to detect CA from standard 12-lead ECGs, achieving an AUC of 0.91 and predicting CA over six months before clinical diagnosis in 59% of patients. However, it was limited by single-center data and uncertainty regarding cardiac involvement [28]. Harmon et al. validated this AI-ECG tool with an AUC of 0.84, though performance was lower for the Hispanic population [29].

In echocardiography, Zhuang et al. used the You Only Look Once, Version 3′ model (YOLOv3 model) for superior segmentation accuracy of left-ventricle ultrasound images [30]. Chao et al. employed the ResNet50 model, a deep learning (DL) network on the basis of transthoracic echocardiography (TTE), to differentiate CA from constrictive pericarditis (CP), achieving an AUC of 0.97, though limited by a small sample size [31]. Li et al. developed a DL framework to differentiate increased left ventricle (LV) wall thickness etiologies, with AUCs ranging from 0.73 to 0.94, yet faced potential referral bias [32].

In CMR studies, Agibetov et al. used Convolutional Neural Networks (CNNs) to recognize imaging patterns of CA, achieving an AUC of 0.96, but most patients had advanced HF [33]. Eckstein et al. (2021) utilized ML algorithms for multi-chamber strain and cardiac function, attaining an AUC of 0.960 [34]. Xu et al. (2022) implemented an ML named progressive sequential causal generative adversarial network (PSCGAN) to synthesize late gadolinium enhancement (LGE)-equivalent images from cine MRI, with a segmentation accuracy of 97.17% [35].

Scintigraphy studies by Halme et al. used CNN to classify scintigraphy images, achieving an AUC of 0.94 and 99% accuracy for Perugini grade classification [36]. Delbarre et al. developed a DL model for detecting significant cardiac uptake on scintigraphy with 98.9% sensitivity and 99.5% specificity [37].

Pathology studies by Palstrøm et al. used a Support Vector Machine (SVM) algorithm to identify amyloid signature proteins in biopsies with 100% accuracy [38]. Kim et al. used Raman spectroscopy to detect amyloidosis in renal tissue, achieving 95.6% to 98.4% accuracy [39]. Wang et al. used an ML approach to identify the depositions of amyloid in histological slides [40], and Kessel et al. developed a DL algorithm for detecting amyloid deposits in corneal tissue with 86% sensitivity and 92% specificity [41].

Lastly, Garofalo et al. (2022) applied an ML approach to predict the monoclonal light chain toxicities in serum studies, achieving an AUC of 87% [42], while David et al. developed an ML model to predict amyloidogenicity in immunoglobulin sequences with the accuracy of prediction around 60.84% to 81.08% [43]. These studies highlight the potential of AI in enhancing the accuracy and early diagnosis of CA across various other diagnostic modalities, though further validation and larger-scale studies are needed. The summaries of the studies on AI and CA have been illustrated in Table 1.

**Table 1:** Summaries of the studies on the role of AI in diagnosing CA AI: Artificial Intelligence; AUC: Area Under the Curve; CA: Cardiac Amyloidosis; CP: Constrictive Pericarditis; CMR: Cardiac Magnetic Resonance; CNN: Convolutional Neural Network; DL: Deep Learning; ECG: Electrocardiogram; HF: Heart Failure; ML: Machine Learning; SVM: Support Vector Machine; ATTR-CA: Transthyretin Cardiac Amyloidosis; AL-CA: Immunoglobulin Light Chain Amyloidosis; YOLOv3: ’You Only Look Once, Version 3′ model; MRF: Markov Random Field model

Study	Year	AI Application	Description	Sample Size	Key Findings	Limitations
Tison et al [26]	2017	ECG	ML algorithms for cardiac parameters estimation using 36,186 ECGs	36,186	AUC of 0.86 for predicting CA	Single-center, optimized for normal sinus rhythm only
Goto et al [27]	2021	ECG	A model of AI-ECG for identifying CA using 10,933 ECGs	10,933	AUCs of 0.85 to 0.91, capable of identifying CA before clinical diagnosis	Probability of undetected cases in the control group, false labels
Grogan et al [28]	2021	ECG	AI-based tool for diagnosing CA from standard 12-lead ECG	2,541	AUC of 0.91 predicted CA over six months before clinical diagnosis	Single-center uncertainty regarding cardiac involvement in some patients
Harmon et al [29]	2022	ECG	Follow-up validation study of AI-ECG tool	N/A	AUC of 0.84, acceptable performance across various subgroups	Lower AUC for the Hispanic population
Zhuang et al [30]	2022	Echocardiography	YOLOv3 model for segmenting left-ventricle ultrasound images	N/A	Superior segmentation accuracy, robustness, and computational speed	N/A
Chao et al [31]	2022	Echocardiography	ResNet50 model for differentiating CA from CP	381	AUC of 0.97 for differentiating CP from CA	Small sample size, single-center
Li et al [32]	2022	Echocardiography	DL framework to differentiate the etiologies of increased LV wall thickness	586	AUCs of 0.73 to 0.94, the final fusion model outperformed individual view-dependent models	Single-center, retrospective nature, potential referral bias
Agibetov et al [33]	2021	CMR	CNNs to recognize imaging patterns associated with CA	502	AUC of 0.96, 94% sensitivity, 90% specificity	Most patients had advanced HF, and may not identify early disease
Eckstein et al [34]	2021	CMR	ML algorithms using multi-chamber strain and cardiac function	N/A	AUC of 0.960, competitive diagnostic accuracies	Unmatched retrospective cohort, unsupervised algorithmic model
Xu et al [35]	2022	CMR	PSCGAN to synthesize LGE-equivalent images from cine MRI	N/A	Segmentation accuracy of 97.17%, SSIM of 0.78 for synthesized images	Small dataset, reliance on manual segmentation for ground truth, and potential overfitting and training instability
Halme et al [36]	2022	Scintigraphy	CNN to automatically identify and classify the images in scintigraphy	1,334	AUC of 0.94, accuracy of 99% for Perugini grades classification	Dateset imbalance, few positive cases, imaging protocl variability, lack of clinical validation, potential overfitting
Delbarre et al [37]	2022	Scintigraphy	DL model to detect significant cardiac uptake on scintigraphy	4,681	Sensitivity of 98.9%, specificity of 99.5%	Cannot differentiate the uptake related to amyloidosis and non-amyloidosis
Palstrøm et al [38]	2022	Pathology	SVM algorithm to detect amyloid signature proteins in biopsies	153	Effective categorization based on subtypes with an accuracy of 1.0	N/A
Kim et al [39]	2022	Pathology	Raman spectroscopy for amyloidosis detection	N/A	Accuracy ranging from 95.6%-98.4% compared with histopathology	Small sample size
Wang et al [40]	2022	Pathology	ML approach to identify amyloid depositions in histological slides	154	Model comparable to the gold standard of manual segmentation	N/A
Kessel et al [41]	2022	Pathology	DL algorithm to detect and quantitate amyloid deposits in corneal tissue	42	Sensitivity of 86%, specificity of 92%, F-score of 81	N/A
Garofalo et al [42]	2022	Serum Studies	ML approach to predict the toxicity of monoclonal light chains	1,075	Sensitivity of 76%, specificity of 82%, AUC of 87%	Limited sample sizes
David et al [43]	2022	Serum Studies	ML model to predict amyloidogenicity in immunoglobulin sequences	143	Prediction accuracies ranging 60.84% - 81.08%	Limited sample sizes

Staging systems for prognosis

For TTR-CA, two staging systems have been proposed [44,45]. The first staging systems for ATTRw-CA were developed based on cardiac troponin and N-terminal pro-B-type natriuretic peptide. The troponin T and NT-proBNP thresholds are 0.05 ng/mL and 3000 pg/mL, respectively. For stage I (both values below cutoff), stage II (one value above), and stage III (both values above), the corresponding four-year overall survival rates were 57%, 42%, and 18%, respectively [45]. The second staging system, verified in patients with ATTRwt or ATTRv, is based on the estimated glomerular filtration rate (eGFR) and serum levels of NT-proBNP. The thresholds for eGFR and NT-proBNP are 45 mL/min per 1.73 m² and 3000 pg/mL, respectively. Stage I is described as eGFR ≥45 mL/min per 1.73 m² and NT-proBNP ≤3000 pg/mL, Stage III as eGFR <45 mL/min per 1.73 m² and NT-proBNP >3000 pg/mL, and the remainder fall into Stage II. The corresponding median survival times were 69.2 months, 46.7 months, and 24.1 months for Stage I, Stage II, and Stage III patients, respectively [44]. The revised Mayo staging system proposed for AL-CA is based on NT-proBNP, cardiac troponin T, and the difference between involved and uninvolved serum-free light chains (dFLC). The thresholds for NT-proBNP, cardiac troponin T, and dFLC are 1800 ng/L, 0.025 mcg/L, and 18 mg/dL, respectively. In Stage I, all factors are within the normal limit; in Stage II, any factor is high; in Stage III, any two factors are high; in Stage IV. Stage I has a median survival of 55 months and a five-year survival rate of 50%. For patients undergoing stem cell transplantation, median survival is not reached with a four-year survival rate of 87%. Stage II has a median survival of 19 months and a five-year survival rate of 35%, with patients undergoing stem cell transplantation having a median survival of 97 months and a four-year survival rate of 72%. Stage III has a median survival of 12 months and a five-year survival rate of 20%, with stem cell transplantation patients having a median survival of 58 months and a four-year survival rate of 56%. Stage IV has a median survival of five months and a five-year survival rate of 15%, with stem cell transplantation patients having a median survival of 22 months and a four-year survival rate of 46% [46-48]. 

Management

In treating cardiac amyloidosis, volume control is essential and frequently involves loop diuretics and mineralocorticoid antagonists. Use of thiazide diuretics should be done with caution to prevent kidney problems because of diastolic dysfunction and restrictive physiology [19]. Kidney function strongly influences results, and prognosis can deteriorate with higher dosages of diuretics [44]. Because cardiac amyloidosis causes specific physiological changes, such as decreased stroke volume and a reliance on heart rate to sustain cardiac output, standard heart failure treatments are generally not advised. Frequently, patients have poor tolerance to beta-blockers, angiotensin inhibitors, and neprilysin inhibitors. However, spironolactone and other mineralocorticoid antagonists have demonstrated some advantages in lowering hospitalizations and cardiovascular mortality in individuals with amyloidosis [49]. Theoretically, sodium-glucose cotransporter inhibitors may help HFpEF, yet there is little data on cardiac amyloidosis [50].

Tafamidis is the only FDA-approved treatment for TTR amyloidosis, a TTR stabilizer demonstrated in the ATTR-ACT trial to decrease mortality and cardiovascular hospitalizations. Additionally, it lowers the deterioration in life quality and functional ability. Tafamidis is well-tolerated with a favorable side-effect profile despite its high annual cost of $225,000. In contrast, there are alternatives, such as diflunisal, which are less well-tolerated and need more substantial evidence. The benefits of advanced diseases diminish with time, so early diagnosis and treatment are critical. Programs for copayment assistance are frequently required to enable patients to afford tafamidis [51]. 

TTR silencers are approved by the FDA for the treatment of ATTRv polyneuropathy and are currently being tested for the treatment of ATTR-CM. Patisiran and vutrisiran are types of small interfering RNA therapeutics, while inotersen is an antisense oligonucleotide therapeutic. These treatments are known to work effectively when used in combination with stabilizers, but there is still limited data on the effectiveness of combined therapy [19]. In laboratory studies, TTR disrupters such as doxycycline and epigallocatechin-3-gallate have shown promise in reversing amyloid deposition in affected organs; however, their effectiveness has not been widely studied, and they are no longer recommended as standard care [52,53]. Antifibril antibodies designed to trigger the immune system for amyloid resorption have been developed, but their effectiveness has yet to be conclusive as they are either still in clinical trials or have failed to meet study endpoints. An emerging therapy involves gene editing using clustered regularly interspaced short palindromic repeats (CRISPR) and the CRISPR-associated protein 9 (Cas9) endonuclease complex, which permanently modifies genomic DNA. A phase 1 clinical study showed a sustained suppression of TTR production after a single infusion, with no significant adverse events reported during short-term follow-up [54].

AF is common in cardiac amyloidosis, affecting up to 56% of AL amyloidosis patients and 70% of those with ATTR-CM [55]. A significant management challenge is the high risk of thromboembolism, with up to 33% of patients having intracardiac thrombi, even on anticoagulation. Guidelines recommend anticoagulation for all AF patients, regardless of CHA2DS2-VASc score, and transesophageal echocardiography before cardioversion. There is limited evidence comparing direct oral anticoagulants and warfarin, though the former is commonly used [56]. Left atrial appendage closure devices may be an option for those who cannot take anticoagulants, but data is limited. These are typically associated with low-rate ventricular response, and low-dose beta-blockers can be effective if tolerated. For refractory AF, atrioventricular junctional ablation with pacemaker placement may be considered. Digoxin, once thought contraindicated, may be used with caution for rate control. Amiodarone is usually well tolerated for rhythm control, and catheter ablation is more successful in early-stage disease [19].

Collaborating with a cardiologist who specializes in cardiac amyloidosis can be crucial in several scenarios. Specialists can tailor treatments for patients with progressive heart failure despite optimized volume management and assess advanced therapies. They should closely monitor conduction disease, which is common in these patients, for the potential need for pacemakers and cardiac resynchronization therapy. Ventricular arrhythmia risk stratification is essential, as nonsustained arrhythmias are frequent, though data on survival benefits from implantable cardioverter-defibrillators are inconclusive [19]. In patients with concurrent aortic stenosis and cardiac amyloidosis, particularly older individuals, transcatheter aortic valve replacement can offer symptom relief and survival benefits [57]. Specialists can help the patients by connecting with advocacy groups for support and education. Assessing options for these patients include mechanical circulatory support, heart transplantation, and palliative care. Heart transplantation is contraindicated in the presence of extracardiac manifestations such as neuropathy, malnutrition, and uncontrolled disease (AL amyloidosis). Mechanical circulatory support, including left ventricular assist devices (LVADs), is often poorly tolerated in these patients due to small left ventricular cavity size and biventricular involvement. Palliative care may be an option for symptomatic relief, even if mechanical circulatory support or heart transplantation is the chosen pathway [19].

Nephrologists play a significant role in the multidisciplinary management of ATTR or AL amyloidosis patients who have impaired kidney function from amyloid CM or amyloid nephropathy and in addressing metabolic consequences of chronic kidney disease (CKD), amyloidosis therapy-related nephrotoxic effects, nephrotic syndrome, and cardiorenal syndrome [19].

Genetic testing can determine if ATTR-CM is wild-type or a pathogenic mutation. Sporadic cases are common among patients in non-endemic areas. Approximately 9% of known TTR mutations do not cause amyloidosis. Patients with a family history of neuropathy, CM, or amyloidosis can be referred to a genetic counselor for a three-generation pedigree and to discuss the risks and benefits of genetic testing. Results may identify late-onset diseases and low-penetrance disorders. Ongoing studies are looking at early-onset and subclinical diseases. Counseling also involves reviewing results, discussing family histories, and considering testing for at-risk carriers over 18 years old [58]. Table 2 outlines the management strategies for CA. 

**Table 2:** Management of cardiac amyloidosis NSAID: nonsteroidal anti-inflammatory drug, siRNA: Small interfering ribonucleic acid, HFpEF: heart failure with preserved ejection fraction, CRISPR: clustered regularly interspaced short palindromic repeats, Cas9: CRISPR associated protein 9, AF: atrial fibrillation

Management options	Details
Volume Control	Essential in treating cardiac amyloidosis; Loop diuretics and mineralocorticoid antagonists; Caution with thiazide diuretics to prevent kidney problems; Kidney function influences prognosis; higher diuretic doses can worsen outcomes
Standard Heart Failure Treatments	Generally, not advised due to specific physiological changes; Poor tolerance to beta-blockers, angiotensin inhibitors, neprilysin inhibitors; Spironolactone & other mineralocorticoid antagonists can reduce hospitalizations and cardiovascular mortality; Limited data on sodium-glucose cotransporter inhibitors for HFpEF
FDA-Approved Treatments	Tafamidis (TTR stabilizer); Decreases mortality and cardiovascular hospitalizations; Lowers deterioration in life quality and functional ability; Well-tolerated, favorable side-effect profile; High annual cost ($225,000); copayment assistance often required
Alternatives and Emerging Therapies	Diflunisal (NSAID): Less well-tolerated, needs more evidence. TTR Silencers: Includes patisiran, vutrisiran (siRNA), and inotersen (antisense oligonucleotide) for ATTRv polyneuropathy, limited data for ATTR-CM. TTR Disrupters: Doxycycline and epigallocatechin-3-gallate not recommended as standard care. Antifibril Antibodies: Inconclusive effectiveness, in trials. Gene Editing (CRISPR-Cas9); Promising early clinical studies, sustained suppression of TTR production
Atrial Fibrillation Management	Affects up to 56% of AL amyloidosis and 70% of ATTR-CM patients; High thromboembolism risk; anticoagulation recommended regardless of CHA2DS2-VASc score; Transesophageal echocardiography before cardioversion; Limited evidence comparing direct oral anticoagulants and warfarin; Left atrial appendage closure devices for those who cannot take anticoagulants; Low-dose beta-blockers can be effective if tolerated; If refractory AF: Atrioventricular junctional ablation with pacemaker; Amiodarone for rhythm control
Advanced Disease management	Pacemaker, implantable cardioverter defibrillator for standard indications like conduction disease, ventricular tachycardia/fibrillation. Options for advanced heart failure: mechanical circulatory support, heart transplantation, palliative care. Heart transplantation contraindications: neuropathy, malnutrition, uncontrolled disease (AL amyloidosis). Connect patients with advocacy groups; palliative care for symptomatic relief. Multidisciplinary approach for other systemic manifestations

## Conclusions

CA is a complex and challenging condition characterized by the pathological deposition of amyloid fibrils, which can compromise cardiac function. Advances in understanding the disease's pathophysiology, particularly the role of TTR and AL proteins, have led to improved diagnostic and therapeutic strategies. The use of AI in diagnostic processes has shown promise in enhancing the accuracy and speed of disease detection, potentially leading to earlier and more effective interventions. Current management options, including volume control and targeted pharmacotherapies, offer significant benefits but are limited. The high cost of treatments like tafamidis and the need for copayment assistance programs highlight ongoing barriers to optimal care. Furthermore, emerging therapies such as TTR silencers, gene editing technologies, and antifibril antibodies require extensive research to establish efficacy and safety. There is a pressing need for continued research into novel therapeutic approaches, the refinement of AI diagnostic tools, and the development of cost-effective treatments to improve the prognosis and quality of life for patients with cardiac amyloidosis.
